# A randomised, double-blind, placebo-controlled, multicentre clinical trial of AZD1656 in diabetic patients hospitalised with COVID-19: The ARCADIA Trial - implications for therapeutic immune modulation

**DOI:** 10.1016/j.eclinm.2022.101604

**Published:** 2022-08-18

**Authors:** Jamie Chorlton, Zoe Hollowood, Carlene Dyer, Donna Lockhart, Pascal Boekman, Kieran McCafferty, Pete Coffey, Federica Marelli-Berg, John Martin

**Affiliations:** a St George Street Capital, (UK Registered charity No: 1171470), London, UK; b William Harvey Research Institute, Queen Mary University of London, London, UK; c Department of Nephrology, Barts Health NHS Trust, London, UK; d Institute of Ophthalmology, University College London, London, UK; e Division of Medicine, University College London, London, UK

**Keywords:** COVID-19, SARs-COV-2, AZD1656, Glucokinase activator, Diabetes, Regulatory T cells, Tregs, Immune, Clinical trial, ARCADIA, Cytokine, Immunophenotyping

## Abstract

**Background:**

A potential immunotherapeutic role for AZD1656 (a glucokinase activator) in the treatment of COVID-19 was hypothesized. The ARCADIA trial investigated the safety and efficacy of AZD1656 in diabetic patients admitted to hospital with COVID-19.

**Methods:**

The ARCADIA trial was a Phase II randomised, double-blind, placebo-controlled clinical trial. Adult diabetic patients, admitted with COVID-19, were recruited at 28 hospitals in the UK, Romania and Czech Republic and randomly assigned (1:1) to receive AZD1656 tablets (100mg twice a day), or matched placebo, for up to 21 days, in addition to usual care. All involved were masked to treatment allocation. The primary endpoint was clinical improvement measured at Day 14. The Full Analysis Set (FAS) included all patients who received at least one dose of assigned treatment. ARCADIA is complete and registered with ClinicalTrials.gov (NCT04516759).

**Findings:**

Between 29 September 2020 to 16 April 2021, 170 patients were screened and 156 patients were randomised, three of whom did not commence treatment. Of the remaining 153, 80 were assigned to AZD1656 and 73 were assigned to placebo and included in the Full Analysis Set (FAS). The primary analysis showed no statistically significant difference between groups (AZD1656: 76·3%; Placebo: 69·9%, *p=*0·19). There was no difference in the number of adverse events between groups (AZD1656: 35·7%; Placebo: 33·3%). Mortality was lower in the AZD1656 group compared to the placebo group (AZD1656: four (5%); Placebo: nine (12·3%), *p=*0·090)). At Day 7 there were zero deaths in the AZD1656 group compared to six deaths in the placebo group (*p=*0·011, post hoc). A difference between groups in time to hospital discharge was also seen (*p=*0·16). Immunophenotyping data suggested that AZD1656-treated patients had a less pro-inflammatory immune response and a better adaptive immune response than those treated with placebo.

**Interpretation:**

Although the trial did not achieve its primary endpoint, AZD1656 was associated with a decrease in deaths and a reduction in the duration of hospitalisation, as compared to Placebo. Immunophenotyping and immunochemistry indicated an immunomodulatory effect of AZD1656. The trial suggests a beneficial therapeutic effect of AZD1656 and identifies a new therapeutic concept: small molecule activation of endogenous homeostatic immune cells which themselves become the therapeutic agent within the body. Phase 2 trials of this size carry the risk of false positive results and confirmation of these results in a larger clinical trial is now required.

**Funding:**

UK Research and Innovation (UKRI) ‘Innovate UK’ programme and Excalibur Medicines Ltd.

**Research in context**

**Evidence before the Study**

Between March 2020 and June 2020, multiple searches of the available open access literature and of clinicaltrials.gov using the terms “COVID-19” and SARs-CoV-2” were conducted, which confirmed that there were no approved treatments for COVID-19 when this trial was designed and that an inappropriate immune response including “cytokine storm” was responsible for the pathophysiology of people with severe COVID-19. Migration of Tregs to sites of inflammation was known to be essential for their homeostatic function, a process which requires the activation of glycolysis by the enzyme glucokinase (also known as Hexokinase IV). AZD1656 (a specific glucokinase activator) had shown short-term efficacy in reducing blood glucose levels in people with type 2 diabetes and reduced the risk of skin transplant rejection in AZD1656-fed rodents, but its immune effects in humans and potential to treat COVID-19 humans was unknown.

**Added value of this study**

This study increases our understanding of COVID-19 and in particular the immune system response to COVID-19, by examining both immunochemistry and immunophenotyping data in diabetic patients hospitalised with this disease. The study increases our understanding of the drug AZD1656 and specifically, its potential to treat COVID-19 and/or other illnesses where an aberrant or unwanted T-cell mediated immune response is involved in the pathophysiology of the disease.

**Implications of all the available evidence**

Although the trial did not achieve its primary endpoint, AZD1656 was associated with a reduction in mortality (*p=*0.090) and a reduction in hospitalization time (*p=*0.16) in people with diabetes admitted to hospital with COVID-19. AZD1656 appeared to induce a measurable immunomodulatory effect, consistent with an active rebalancing of the immune response, which could explain the improved clinical outcomes seen in AZD1656-treated patients. AZD1656 may benefit patients with other conditions where an inappropriate T-cell mediated immune response is involved, including respiratory viral infections, organ transplant rejection and those with T-cell mediated auto-immune conditions.

Alt-text: Unlabelled box

## Introduction

SARs-COV-2 induces an excessive proinflammatory response in severely ill patients, resulting in high concentrations of pro-inflammatory cytokines and suppression of anti-inflammatory Regulatory T (Treg) cells, leading to a poor prognosis from COVID-19.^1^^,^^2^ Both type 1 (T1DM) and type 2 diabetes mellitus (T2DM) confer an increased risk of mortality^3^; moreover, poor glycaemic control (as defined by an elevated HbA1c level) is an independent predictor of increased mortality in the disease.^4^

It was hypothesised that AZD1656 (a glucokinase activator) could be used as an immune regulatory medicine to benefit diabetic patients with COVID-19 by both immunomodulation and by controlling the abnormal glucose variability observed in this disease.

AZD1656 had been investigated in 25 prior clinical trials as part of AstraZeneca's Type 2 diabetes development program, with over 960 patients exposed to AZD1656 (for up to 6 months treatment duration). It was well-tolerated in patients with type 2 diabetes and reduced blood glucose but only for 3-4 months, thus limiting its long-term use for patients with diabetes.^5^^,^^6^

In vitro studies demonstrated that migration of regulatory T cells (Tregs) to inflamed tissue is crucial for their immune-modulatory function; a process that is regulated by glucokinase-dependent glycolysis.^7^ The specific dependence of Treg migration on glucokinase-mediated glycolysis had been demonstrated in humans where Tregs, but not conventional T cells, from carriers of a loss of function gene polymorphism in GCK regulatory protein (GKRP) displayed enhanced motility ex vivo. Carriers of the polymorphism displayed decreased circulating Tregs suggesting increased localisation in tissues. Furthermore, in-vivo studies using Tregs as an interventional treatment through adoptive transfer have shown benefit in an acute lung injury model through mediating the resolution of lung injury.^8^ Glycolysis also plays an important role in the function of pro-inflammatory T effector cells (Teffs), however Teffs migrate via activation of Hexokinase I (HKI), but not glucokinase (HKIV). AZD1656 is a specific activator of glucokinase with >100-fold effect compared to HKI and would therefore be expected to enhance selectively Treg cell migration.^9^

Uncontrolled (or unwanted) innate and adaptive immune responses might therefore be controlled with a glucokinase activator (such as AZD1656) by specifically enhancing the migration of Treg cells to inflammatory sites, thereby reducing inflammation and helping to restore immune homeostasis. In COVID-19, an effective treatment of this type could eliminate the most damaging effects of the disease, leading to reduced cardiorespiratory complications resulting in decreased hospitalization times and reduced mortality.

The aim of this trial was to investigate the use of AZD1656 in the treatment of patients with diabetes hospitalised with COVID-19. The primary objective of the study assessed clinical outcome at Day 14, using the WHO 8-point Ordinal Scale for Clinical Improvement (OSCI) rating system. The secondary objectives focussed on mortality, duration of hospitalisation, glycaemic control, and safety. The exploratory objectives included an assessment of the immunological effects of the drug.

## Methods

### Study Design

The ARCADIA Trial was a double-blind, randomised, placebo-controlled, clinical trial to evaluate AZD1656 in the treatment of diabetic patients hospitalised with COVID-19. The trial was conducted at 28 hospitals in the UK, Romania and the Czech Republic. The trial was sponsored by the charity St George Street Capital. It was performed in accordance with the principles of the International Conference on Harmonisation Good Clinical Practice guidelines and approved by the UK Medicines and Healthcare products Regulatory Agency. Ethical approval was obtained from the East Midlands-Leicester South Ethics Committee (REC 20/EM/0198) in the UK, from the National Bioethics Committee of Medicines and Medical Devices in Bucharest, Romania, and from the Multicentre Ethics Committee of the Institute of Clinical and Experimental Medicine and Thomayer's Hospital in Prague, the Czech Republic. The full protocol and statistical analysis plan are publicly available on ClinicalTrials.gov (NCT04516759).

### Participants

Patients were eligible if they had either type 1 or type 2 diabetes and had been hospitalised with suspected or confirmed COVID-19 and a blood glucose level at or above 4 mmol/L. Local investigators recruited participants admitted to their hospital. 156 patients aged 18 or older took part in the trial. Written informed consent was obtained from all patients.

### Randomisation and masking

Eligible patients were randomly assigned (1:1) to either the AZD1656 (plus usual care) group or to the placebo (plus usual care) group. Randomisation was performed by the investigators using a centralized computer-based randomisation system. Treating staff, patients and study team were blinded to the treatment intervention. Randomisation blocks were prepared by a statistician not involved in the trial using SAS software. Matching active and placebo tablets were packaged in identical bottles (distinguishable only by a unique bottle number) in order to achieve blinding.

### Procedures

AZD1656 or placebo tablets were taken orally, twice per day, at approximately the same time each day with or just after meals. 50mg film-coated tablets of AZD1656 (or matching placebo) were dosed at 100mg BID (a total daily dose of 200 mg). Patients received study treatment for up to a maximum of 21 days, or until date of discharge from hospital, or date of transfer to mechanical respiratory ventilation or date of death. The day on which the patient discontinued study drug was defined as Study Drug Discontinuation (SDD). Therefore, SDD could occur at any timepoint between Day 2 and Day 21. Patients also received standard of care for COVID-19, which was determined at an individual site and patient level. All treatments were recorded as concomitant medications. Patients were assessed daily to capture their WHO OSCI status. Safety assessments included daily monitoring for adverse events, clinical laboratory testing and daily vital signs measurements. Analysis of NT-proBNP and hsTroponin (hs-CTNT) was performed at screening and the final study visit to determine the extent of cardiac injury in patients receiving AZD1656 versus placebo. A final visit took place 7 days after completion of study treatment to capture any new safety information or changes to concomitant medication. Immunophenotyping and immunochemistry panels were conducted to explore the effect of AZD1656 on specific immune characteristics. Immunophenotyping was conducted by Flow Cytometry: between group comparison (AZD1656 versus placebo) of levels of T, B and NK cells including specific Treg and memory T cell populations. (See Supplementary Material for a summary of the immunophenotyping methodology.) Immunophenotyping was performed at Queen Mary University of London (QMUL), UK. Immunochemistry was conducted using the MSD U-Plex multiplex assay for assessment of the following biomarkers: G-CSF, GM-CSF, IL-1B, IL-4, IL-6, IL-8, IL-10, IL-12, and MIP-1a. PK analysis and immunochemistry were performed at York Bioanalytical Solutions, UK. Clinical data were captured in an electronic Case Report Form (Medidata RAVE). The trial was monitored by a Clinical Research Organisation (Clinipace Inc). The Chief Investigator was Dr Kieran McCafferty, Barts Health NHS Trust, UK.

### Outcomes

The primary outcome was Clinical Improvement assessed centrally as the percentage of subjects who were ‘responders’ (patients with WHO OSCI score 1, 2 or 3 at Day 14), comparing AZD1656 treatment to placebo. To check the robustness of the primary efficacy analysis (which focussed on a single timepoint), a longitudinal analysis based on the WHO OSCI path from Day 1 to Day 14 was also conducted.^10^ To explore if subgroups affected clinical outcome, the primary efficacy analysis on clinical improvement was repeated for subgroups of the following variables: Vitamin D group, Race, Sex. Age group, Diabetes type and Site. Secondary and safety outcome measures included clinical improvement (measured as the proportion of patients in each WHO OSCI category at day 7, 14 and 21 post randomisation); degree of glycaemic control; time from admission to hospital discharge; all-cause mortality (Day 28), time from admission to mechanical respiratory ventilation, proportion of serious adverse events and proportion of treatment emergent adverse events leading to study drug discontinuation. Predetermined exploratory comparisons of immunophenotyping and immunochemistry parameters over time were made for each treatment group with the baseline values for that patient subset, for changes from baseline within each group and between groups at each timepoint. Levels of 25-hydroxyvitamin D levels were measured before treatment to compare to clinical outcomes.

Post hoc analyses were performed to examine the trial data in light of the improvements in standard of care and resultant patient outcomes that evolved during the pandemic. Time to hospital discharge became a clinically important measure for health utilisation assessment and several post hoc analyses were performed on this endpoint. The following subgroups of patients were analysed based upon the assumption that certain risk factors could affect COVID-19 outcome: those with high baseline IL-6 (≥ 13 pg/mL), baseline Vitamin D status (< 25 nmol/L and ≥25nmol/L), dexamethasone use, BMI values (≥ 35 mg/m^2^), and baseline Treg levels (< 10·5% or ≥ 10·5%). The day 7 (168 hour) timepoint was identified a priori in the protocol as a secondary endpoint measure for efficacy. It was therefore selected as a suitable timepoint for post hoc analysis of both mortality rate and hospital discharge rate. Mortality was also assessed at study treatment discontinuation (up to Day 21). A post hoc analysis was performed for patients with T2DM taking insulin on hospital admission to determine if there was an increase in insulin requirements during the study. A logistic regression analysis of clinical improvement was also performed post hoc to determine whether any demographic factors or subgroups could be attributed as causal for clinical improvement.

### Statistical analysis

The trial was designed to include 150 randomly assigned patients to AZD1656 or placebo for an estimated total of 75 evaluable patients per group. A two group χ² test with a 5% two-sided significance level would have 76·74% power to detect the difference between a placebo proportion of 0·6 and an AZD1656 proportion of 0·8 when the sample size is 150 (75/group). The Full Analysis Set (FAS) or intention-to-treat population included all 153 randomised patients who received at least one dose of assigned treatment. The primary efficacy analysis was performed on the FAS. To check the robustness of the primary efficacy analysis which focussed on a single timepoint (Day 14), a longitudinal analysis based on the WHO OSCI path from Day 1 to Day 14 was conducted. whereby the completed WHO OSCI paths of patients were converted into a single number (Worth parameter) via pair comparisons on a daily basis using a Bradley-Terry model.^10^ In order to assess clinical improvement using different assumptions from those in the FAS analysis, the primary efficacy analysis was also performed using the per protocol (PP) set. Clinical improvement was compared between both treatment groups using a logistic regression with the canonical logit-link. The primary endpoint and all laboratory results were tested on the two-sided significance level of 5.0%. All other results were tested on the one-sided significance level of 2.5%. An ANOVA was performed on multiple factors including treatment group, sex, age group, race, diabetes type, vitamin D group, and site to confirm the result of the primary efficacy analysis and to explore the impact of the other factors. The secondary efficacy analyses were performed on the FAS and displayed descriptively. Time from hospital admission to hospital discharge (in hours) and time to death in patients receiving AZD1656 compared with placebo was analysed with Kaplan–Meier estimates. All safety endpoints were analysed by descriptive statistics on the Safety Analysis Set (SAF). Statistical analyses were done using SAS software, version 9·4. A Safety Review Committee (SRC) consisting of clinical and other experts was established by the Sponsor to review safety findings during the study and to help ensure patient safety. The trial is registered with ClinicalTrials.gov (NCT04516759) where the full Statistical Analysis Plan can be viewed.

### Role of the funding source

The funders of the trial had no role in study design, data collection, data analyses, data interpretation or writing of the report.

## Results

**Figure 1:**
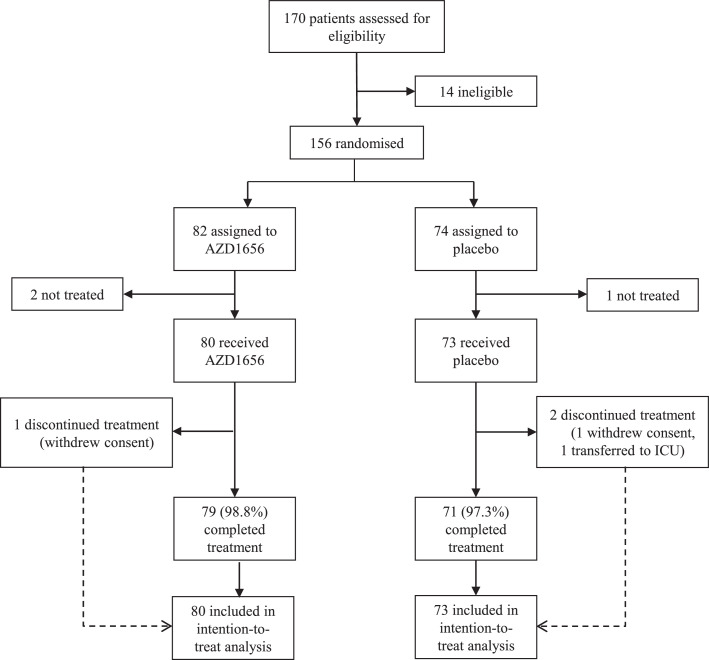
**Trial profile**. 170 patients consented and were screened for study eligibility. 156 eligible patients were subsequently randomised (UK: 74 patients; Czech Republic: 49 patients; Romania: 33 patients). 153 of the randomised patients received at least one dose of either AZD1656 or placebo and were included in the Full Analysis Set (FAS) (the Intent-to-Treat population). The FAS included 80 patients in the AZD1656 group and 73 patients in the Placebo group.

Between 29 September 2020 to 16 April 2021, 170 patients were screened and 156 of these were randomised across 3 European countries (UK: 74 patients; Czech Republic: 49 patients; Romania: 33 patients). 7 patients who were randomised with suspected COVID-19 did not subsequently test positive via PCR and remained in the trial, as allowed per protocol. Of the 156 randomised patients, 153 were treated with AZD1656 (80 patients) or placebo (73 patients) and included in the Full Analysis Set (FAS) (Figure 1). Male and female patients aged 18 years and older with T1DM or T2DM, who were hospitalised with suspected or confirmed COVID-19 categorised as stage 3, 4 or 5 on the WHO OSCI and who were not expected to progress to intubation or mechanical ventilation within the next 24 hours, were included.

Almost all patients completed treatment according to the protocol (AZD1656: 98·8%; Placebo: 97·3%). All had blood glucose levels > 8·5 mmol/L at enrolment (Table 1). The mean age was similar between groups (AZD1656: 63·6 years; Placebo: 65·0 years) and the ratio of men to women was approximately 2:1. Most patients were white (AZD1656: 85·0%; Placebo: 90·4%). Mean duration of treatment (not shown) was similar in both groups (AZD1656: 7·1 days; Placebo: 7·6 days).

**Table 1:** Baseline characteristics.

	Placebo (*N* = 73)	AZD1656 (*N* = 80)
Sex		
Female	26 (35·6%)	30 (37·5%)
Male	47 (64·4%)	50 (62·5%)
Age group		
≥ 18 and < 65 years	31 (42·5%)	38 (47·5%)
≥ 65 and < 85 years	41 (56·2%)	41 (51·3%)
≥ 85 years	1 (1·4%)	1 (1·3%)
Mean (SD)	65·0 (11·71)	63·6 (11·44)
Race		
Asian	5 (6·8%)	7 (8·8%)
Black or African American	0	4 (5·0%)
White	66 (90·4%)	68 (85·0%)
Other	2 (2·8%)	1 (1·3%)
BMI (kg/m2)		
Mean (SD)	34 (7·08)	32·8 (6·22)
HbA1c (%)		
Mean (SD)	6·6 (2·04)	6·9 (2·43)
Blood glucose group at baseline		
4.0 - 8.5 mmol/L	0	0
>8.5 mmol/L	73 (100%)	80 (100%)
Diabetes type		
Type 1	2 (2·7%)	1 (1·3%)
Type 2	71 (97·3%)	79 (98·8%)
Vitamin D group		
< 25 nmol/L	27 (37·0%)	31 (38·8%)
≥ 25 nmol/L	45 (61·6%)	46 (57·5%)
Missing	1 (1·4%)	3 (3·8%)
Received Prior Medications		
At least one medication	44 (60.3%)	49 (61.3%)
Biguanides	12 (16.4%)	16 (20%)
WHO OSCI Rating		
3 - Hospitalised, no oxygen	13 (17·8%)	19 (23·8%)
4 - Hospitalised, oxygen via mask or nasal prongs	42 (57·5%)	52 (65·0%)
5 - Hospitalised, Non-invasive ventilation or high flow oxygen	18 (24·7%)	9 (11·3%)

Data are n (%), mean (SD); N = total number of patients; BMI = Body Mass Index; WHO OSCI = World Health Organisation Ordinal Scale for Clinical Improvement

The proportion of patients receiving prior medications (i.e., stopped before study medication was started) was similar between groups for all medications (AZD1656: 61·3%; Placebo: 60·3%). The most frequently reported prior medications were biguanides (AZD1656: 20·0%; Placebo: 16·4%).

In accordance with the inclusion criteria for the study, suspected or confirmed COVID-19 and T1DM or T2DM were reported for all patients. Most patients had T2DM (150/153) with only three patients with T1DM. Of the concomitant medical conditions considered to be risk factors for COVID-19 (Table 2), obesity was the most frequently reported (AZD1656: 26·3%; Placebo: 34·2%) followed by asthma (AZD1656: 12·5%; Placebo: 12·3%) and COPD (AZD1656: 5·0%; Placebo: 11·0%).

**Table 2:** Summary of concomitant medical conditions considered to be risk factors for COVID-19 (FAS).

Preferred Term	Placebo (*N* = 73)	AZD1656 (*N* = 80)
Obesity	25 (34·2)	21 (26·3)
Asthma	9 (12·3)	10 (12·5)
COPD	8 (11·0)	4 (5·0)
Myocardial ischaemia	5 (6·8)	6 (7·5)
Left ventricular dysfunction	4 (5·5)	2 (2·5)
Cardiac failure congestive	2 (2·7)	1 (1·3)
Acute myocardial infarction	2 (2·7)	0
Cardiac failure	1 (1·4)	1 (1·3)
Cardiac failure chronic	2 (2·7)	0
Coronary artery disease	1 (1·4)	1 (1·3)
Myocardial infarction	1 (1·4)	1 (1·3)
Cerebrovascular accident	1 (1·4)	0
Ischaemic stroke	1 (1·4)	0
Lacunar stroke	0	1 (1·3)

Data are n (%); N = total number of patients; COPD = Chronic Obstructive Pulmonary Disease. FAS = full analysis set; Patients may be counted in more than one category.

Key efficacy outcomes are summarised in Table 3. The primary analysis of clinical improvement, defined as the number of patients who were “responders” (with a WHO score or 1, 2 or 3) at Day 14, showed no statistically significant difference between groups (AZD1656: 76·3%; Placebo: 69·9%; *p=*0·19; RR 1·09 (95% CI 0·67-2·90)). A pre-planned longitudinal analysis showed that AZD1656 performed approximately twice as well as Placebo according to the mean Worth parameter values (AZD1656: 14·8; Placebo: 28·5; *p=*0·038). In a secondary endpoint analysis of clinical improvement at Day 7, 44/80 (55%) of AZD1656 patients were responders, compared to 34/73 (47%) of placebo patients (*p=*0·079). The mortality rate at the end of the study (Day 28) revealed that there were fewer deaths in the AZD1656 group compared to the Placebo group (AZD1656; four (5%): Placebo; nine (12·3%); *p=*0·090).

**Table 3:** Summary of key efficacy outcomes (FAS).

Category or Statistic	Placebo (*N*=73)	AZD1656 (*N*=80)	RR (95% CI)	*p*-value (one sided)
Clinical Improvement^a^ at Day 14	51 (69·9%)	61 (76·3%)	1·09 (0·90, 1·32)	0·19
Longitudinal analysis^b^	28·5	14·8	-	0·038
Clinical Improvement at Day 7	34 (47%)	44 (55%)	-	0.079
Died at any time <28 days (during treatment or follow up)	9 (12·3%)	4 (5·0%)	0·41 (0·13, 1·26)	0·090
Died ≤7 days of start of treatment (post hoc)	6 (8·2%)	0	0·08 (0·00, 1·33)	0·011
Died at any time during treatment (post hoc)	6 (8·2%)	1 (1·3%)	0·15 (0·02, 1·23)	0·045
Discharged from hospital at any time	58 (79·5%)	64 (80·0%)	1·01 (0·86, 1·18)	0·55
Discharged from hospital in first 7 days (post hoc)	18 (24·7%)	30 (37·5%	1·52 (0·93, 2·48)	0·062
Increase in diabetic medication at any time	21 (28·8%)	25 (31·3%)	1·09 (0·67, 1·77)	0·70
Increase in diabetic medication needed ≥ 3 days	13 (17·8%)	12 (15·0%)	0·84 (0·41, 1·73)	0·40
Received intubation/mechanical ventilation	3 (4·1%)	3 (3·8%)	0·91 (0·19, 4·37)	0·61

a Clinical improvement defined as number of treatment responders (WHO score 1, 2 or 3).

b Longitudinal analysis: Worth Parameters for Clinical Improvement from Day 1 to Day 14 (FAS). Data are n(%); N = total number of patients; FAS = Full Analysis Set.

**Figure 2:**
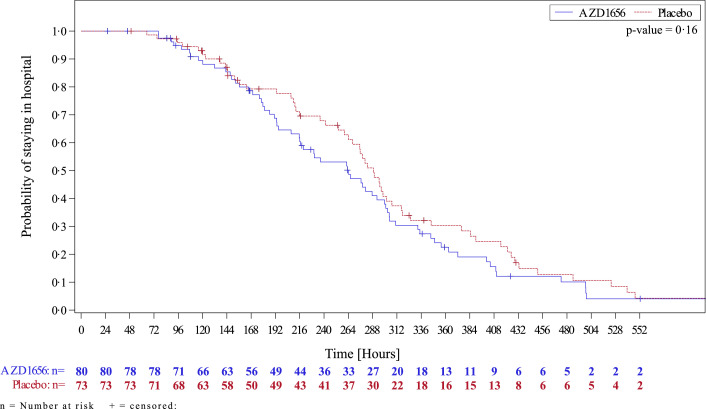
**Time from admission to hospital discharge (analysis set: FAS)**. Kaplan-Meier estimates for time to hospital discharge showed that the probability of staying in hospital curve remained higher in the placebo group than in the AZD1656 group from approximately Day 7 onwards (*p=*0·16).

There was no difference between groups in the proportion of patients ultimately being discharged from hospital (AZD1656: 80·0%; Placebo: 79·5%). Kaplan-Meier estimates for time to hospital discharge showed that the ‘probability of staying in hospital’ curve remained higher in the placebo group than in the AZD1656 group from approximately Day 7 onwards (*p=*0·16) (Figure 2).

Assessment of the patients’ glycaemic control while hospitalised showed no difference between groups in the requirement for additional insulin or additional orally administered agents in patients with T2DM or an increase in insulin dose in patients with T1DM or T2DM. However, consistent with the expected effect of AZD1656, a higher proportion of patients receiving AZD1656 than placebo had glucose levels below Lower Limit of Normal (LLN) during the study (AZD1656: 23·8%; Placebo: 5·5%; *p=*0·0013) (not shown). There were no differences in the proportion of patients who received intubation or mechanical ventilation with three occurring in each group (AZD1656: 3·8%; Placebo: 4·1%; *p=*0·61).

**Figure 3:**
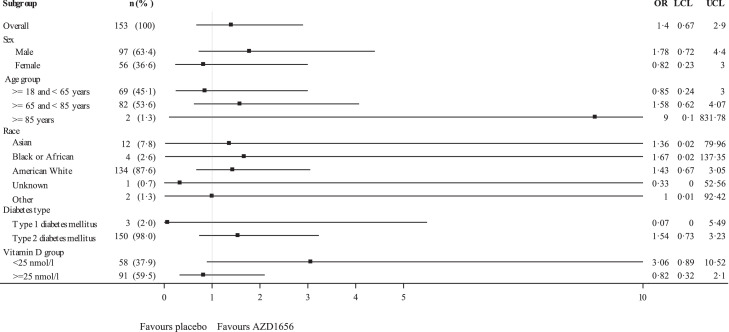
**Clinical improvement at day 14 – odds ratios by overall and subgroups – forest plot (analysis set: FAS)**. Subgroup analyses of clinical improvement at Day 14 indicated a difference between groups in favour of AZD1656 in male patients (OR 1·78; 95% CI 0·72-4·4), patients aged ≥ 65 to < 85 years (OR 1·58; 95% CI 0·62-4·07), and patients with Vitamin D levels < 25 mmol/L (OR 3·06; 95% CI 0·89-10·52).

Subgroup analyses of clinical improvement at Day 14 indicated a difference between groups in favour of AZD1656 in male patients (OR 1·78; 95% CI 0·72-4·4), patients aged ≥ 65 to < 85 years (OR 1·58; 95% CI 0·62-4·07), and patients with low (< 25 mmol/L) baseline Vitamin D levels (OR 3·06; 95% CI 0·89-10·52) (Figure 3).

All patients received at least one concomitant medication during the study and the proportion of patients receiving concomitant medications was similar between groups (see Supplementary Material). The most frequently reported concomitant medications were glucocorticoids, with dexamethasone taken by almost three quarters of all patients AZD1656: 71·3%; Placebo: 75·3%). Some patients were also treated with IL inhibitors which included tocilizumab (AZD1656: 3·8%; Placebo: 4·1%), anakinra (AZD1656: 1·3%; Placebo: none), and sarilumab (AZD1656: 1·3%; Placebo: none).

Key safety outcomes are summarised in Tables 4 and 5. Adverse events (AEs) were reported by a similar proportion of patients between groups (AZD1656: 35·7%; Placebo: 33·3%). A lower proportion of patients in AZD1656 than Placebo reported serious adverse events (AZD1656: 4·8%; Placebo: 10·1%). None of the serious AEs were judged to be related to study treatment. The incidence of adverse events leading to discontinuation from study drug was similar in the 2 groups (AZD1656: 2·4%; Placebo: 2·9%). There was a higher incidence of hypoglycaemia TEAEs in patients receiving AZD1656, all of which were judged as either mild or moderate in severity. There was no difference between groups in thrombotic events throughout the trial and none were considered related to trial drug. There were no other clinically meaningful or significant trends in TEAEs, serious TEAEs, or TEAEs leading to discontinuation. The TEAE profile was consistent with the currently known safety profile of AZD1656 and the underlying diseases. A full list of all TEAEs can be found on ClinicalTrials.gov (NCT04516759). Furthermore, in these patients’ clinical laboratory evaluations, vital signs, physical examination and ECG findings, and chest X-ray/CT scan results were consistent with the nature of the underlying disease and medical history.

**Table 4:** Summary of key safety outcomes (SAF).

Category or Statistic	Placebo (*N*=69)	AZD1656 (*N*=84)	RR (95% CI)	*p*-value (one sided)
All TEAEs	23 (33·3%)	30 (35·7%)	··	··
Serious TEAEs	7 (10·1%)	4 (4·8%)	0·43 (0·14, 1·30)	0·11
Non-serious TEAEs	19 (20·5%)	28 (32·3%)		
TEAEs considered related	1 (1·4%)	9 (10·7%)		
Serious TEAEs considered related	0	0	··	··
TEAEs leading to death	9 (13·0%)	4 (4·8%)		
TEAEs leading to study drug discontinuation	2 (2·9%)	2 (2·4%)	0·77 (0·12, 5·04)	0·59

Data are n(%); n = number of patients with at least one TEAE; TEAE = treatment emergent adverse event; N = total number of patients; SAF = Safety Analysis Set (this includes all patients who received at least one dose of IMP and had at least one post dose safety assessment).

**Table 5:** Summary of serious treatment-emergent adverse events (SAF).

System Organ Class (SOC) Preferred Term	Placebo (*N* = 69, *E* = 36)	AZD1656 (*N* = 84, *E* = 60)	All patients (*N* = 153, *E* = 96)
All SOCs	12 (33·3%)	6 (10·0%)	18 (18·8%)
Cardiac disorders	4 (11·1%)	1 (1·7%)	5 (5·2%)
Angina unstable	0	1 (1·7%)	1 (1·0%)
Cardio-respiratory arrest	1 (2·8%)	0	1 (1·0%)
Cardiogenic shock	1 (2·8%)	0	1 (1·0%)
Myocardial infarction	1 (2·8%)	0	1 (1·0%)
Ventricular tachycardia	1 (2·8%)	0	1 (1·0%)
Gastrointestinal disorders	1 (2·8%)	0	1 (1·0%)
Pancreatitis acute	1 (2·8%)	0	1 (1·0%)
Hepatobiliary disorders	0	1 (1·7%)	1 (1·0%)
Acute hepatic failure	0	1 (1·7%)	1 (1·0%)
Infections and infestations	5 (13·9%)	0	5 (5·2%)
Abscess limb	1 (2·8%)	0	1 (1·0%)
Gastroenteritis	1 (2·8%)	0	1 (1·0%)
Pneumonia bacterial	1 (2·8%)	0	1 (1·0%)
Sepsis	1 (2·8%)	0	1 (1·0%)
Septic shock	1 (2·8%)	0	1 (1·0%)
Injury, poisoning and procedural complications	2 (5·6%)	0	2 (2·1%)
Fall	2 (5·6%)	0	2 (2·1%)
Metabolism and nutrition disorders	0	2 (3·3%)	2 (2·1%)
Dehydration	0	1 (1·7%)	1 (1·0%)
Hypophagia	0	1 (1·7%)	1 (1·0%)
Psychiatric disorders	0	1 (1·7%)	1 (1·0%)
Suicide attempt	0	1 (1·7%)	1 (1·0%)
Vascular disorders	0	1 (1·7%)	1 (1·0%)
Circulatory collapse	0	1 (1·7%)	1 (1·0%)

Data are e(%); e = number of events; TEAE = treatment-emergent adverse event; N = total number of patients; E = total number of TEAEs; SAF = Safety Analysis Set.

Results of pre-planned immunophenotyping and immunochemistry where at least one change of potential biological relevance was observed are summarised in the Supplementary Material.

Differences in the innate immune response were observed between groups. Greater increases in circulating inflammatory monocytes were seen in the Placebo group as compared to the AZD1656 group at all timepoints, with the greatest difference between groups at SDD (*p=*0·079). A correlation was observed between the inflammatory monocyte data and the MIP1α immunochemistry data (a pro-inflammatory cytokine) where greater increases were observed in the Placebo group leading to a difference between groups at SDD (*p=*0·017). A reduction in dendritic cells was observed in the AZD1656 group at the SDD visit (*p=*0·0062), but no difference between groups was detected.

Differences were also observed in the adaptive immune system, particularly in activated (cMet+) T-cells.^11^^,^^12^ Increases in Th1-IFN cMet+ and Th1-TNF cMet+ were observed in the AZD1656 group at multiple timepoints but not the Placebo Group and there was a difference between groups for Th1-IFN cMet+ at one timepoint (SDD) (*p=*0·0049). Differences were observed in the cells involved in the antibody response: Th2 cells decreased in the Placebo group at SDD (*p=*0·035), leading to a difference between groups at SDD (*p=*0·038). B cells also decreased in the placebo group and increased in the AZD1656 group leading to a difference between groups at SDD (*p=*0·045). There was a decrease in levels of Tregs cMet+ (activated Tregs) by Day 11 in both groups and a difference between groups at this timepoint (*p=*0·054). For resting Tregs (those not in the activated state), levels were steady in the Placebo group and fell slightly with AZD1656 treatment with no difference between groups at any timepoint.

**Figure 4:**
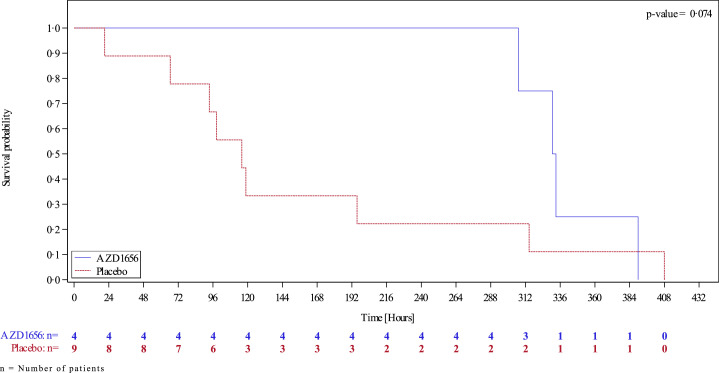
**Time from randomisation to Death - showing deaths only - post hoc (analysis set: FAS)**. More patients died in the Placebo group than in the AZD1656 group (Placebo: 9 AZD1656: 4) and they did so earlier in the course of their hospitalization than those in the AZD1656 group (*p*-value = 0·074). Only patients who died are presented in this plot. All patients, with and without an event (death), were used in the log-rank test.

Post hoc analyses of variables that emerged as important as the pandemic developed generated clinically meaningful findings which need to be tested in a larger trial. An assessment of time from randomisation to death showed that patients in the Placebo group died earlier in the course of their hospitalisation than those in the AZD1656 group (*p=*0·074) (Figure 4). By Day 7 (a pre-determined timepoint for efficacy) there had been six deaths in the Placebo group but none in the AZD1656 group (*p=*0·011, RR 0·08 (0·00, 1·33)) (Table 3). By the time of study treatment discontinuation (up to Day 21) the mortality rate was still lower in the AZD1656 group compared to placebo (AZD1656; one (1·3%): Placebo: six (8·2%) (*p=*0·045, RR 0·15 (0·00, 1·23).

**Figure 5:**
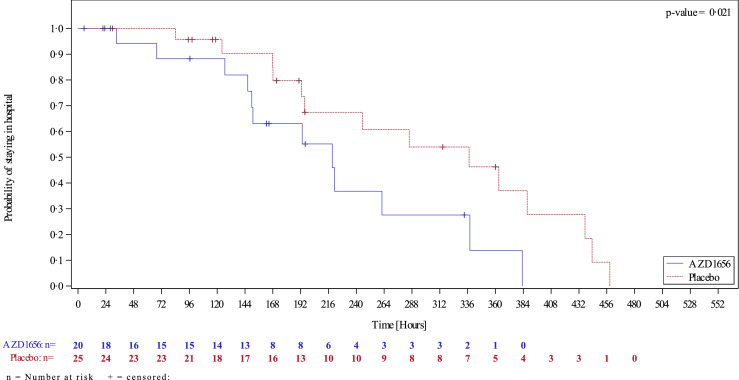
**Time from randomisation to hospital discharge, subgroup**: Patients with baseline IL6 >= 13 pg/mL - post hoc (Analysis Set: FAS) Patients with baseline IL-6 values ≥ 13 pg/mL had a lower probability of staying in hospital in the AZD1656 group than in the Placebo group (*p*-value = 0·021). In this sub-group, the mean hospitalization time was approximately 3 days shorter in the AZD1656 group than the Placebo group (AZD1656: 191 hours; Placebo: 271 hours (*p=*0·056).

Post hoc analysis of 'time to hospital discharge’ showed that the proportion of patients being discharged within 7 days (168 hours) was higher in the AZD1656 group than the Placebo group (37·5% vs 24·7%) (p>0·062, RR 1·52 (0·93, 2·48)). Kaplan Meier estimates of 'time to hospital discharge’ detected differences between treatment groups in certain high risk sub-groups. In patients with low baseline Vitamin D levels (< 25 nmol/L), the ‘probability of staying in hospital’ Kaplan Meier curve was higher in the Placebo group than the AZD1656 group (*p=*0·052) (Supplementary Material). In patients with high baseline IL-6 values (≥ 13 pg/mL), the ‘probability of staying in hospital’ Kaplan Meier curve was also higher in the Placebo group compared to the AZD1656 group (*p=*0·021) (Figure 5) and the mean hospitalization time was 3 days less in the AZD1656 group than in the Placebo group (*p=*0·056). Dexamethasone use, high BMI, or baseline Treg levels did not affected the probability of patients staying in hospital.

A post hoc analysis was performed for patients with T2DM taking insulin on hospital admission to determine if there was an increase in insulin requirements during the study. Results showed that there were no differences between groups. A logistic regression analysis of the baseline factors treatment, sex, age group, race, diabetes type, Vitamin D group and site was conducted post hoc to determine whether any demographic factors or subgroups could be attributed as causal for clinical improvement and this showed no difference between groups.

## Discussion

The trial recruited the planned number of patients and randomisation achieved a well-matched population both demographically and for severity of disease. It was notable that only three patients had T1DM and 150 had T2DM. The study did not achieve its primary endpoint, for reasons that will be discussed, however, an association between AZD1656 treatment and clinical outcome, including reduced mortality, was discovered.

The differences between groups in clinical outcomes supported the trial hypothesis that AZD1656 might be of benefit to patients with diabetes admitted to hospital with COVID-19. The study protocol noted that improved clinical outcomes could be achieved via two potential mechanisms of action: by improved glycaemic control and by modulation of the immune response through enhanced migration of Treg cells. Since no differences in glycaemic control were detected between groups, it is unlikely that the differences in outcomes were a result of the glucose lowering effect of AZD1656. The immunology data however suggested that AZD1656 induced a measurable, immunomodulatory effect, consistent with an active rebalancing of the immune response, which supported the trial hypothesis and could explain the differences in clinical outcomes observed in this trial.

Our understanding of COVID-19, its immunopathology and consequent management of the disease has evolved significantly since this trial began in 2020. Early evidence of adaptive immune dysfunction was derived from autopsy reports of patients with COVID-19 with low numbers of CD8-positive T lymphocytes infiltrating lung tissue.^13^ We now know that mechanisms driving T cell activation and differentiation become dysregulated in severe COVID-19, leading to unfocused T-cell responses which can paralyze the T-cell system preventing appropriate resolution of inflammation following infection.^14^ The ARCADIA trial has generated the first clinical evidence of glucokinase-activated immunomodulation being associated with a reversal of COVID-induced ‘immune-paralysis’ and a restoration of the adaptive immune response. The ARCADIA trial was designed before there were approved therapies for COVID-19. Dexamethasone was subsequently approved and almost three quarters of all study patients in both groups were treated with dexamethasone. Importantly, the differences between groups in both clinical outcomes and immunological measures were additive to the effects of dexamethasone.

Evidence from published data shows that higher Vitamin D levels are not only associated with better COVID-19 outcomes,^15^^,^^16^ but also with higher Treg/total T cell ratios and a more immunosuppressive phenotype.^17^^,^^18^ In this trial, low baseline Vitamin D levels were associated with longer hospitalisation time in the placebo group but not in the AZD1656 group. In those who received AZD1656, mean hospitalisation time was similar in patients with high and low vitamin D, which may reflect a decreased need to use vitamin D by inflammatory cells in the patients treated with AZD1656. A difference was also observed between clinical outcomes and baseline IL-6 levels. Despite being in a higher risk group for a poor outcome from COVID-19,^19^^,^^20^ patients with a high baseline IL-6 (≥ 13 pg/mL) treated with AZD1656 were discharged alive and well from hospital faster than those treated with placebo. Both vitamin D levels and IL-6 levels may be markers to aid stratification of patients in future trials.

**Figure 6:**
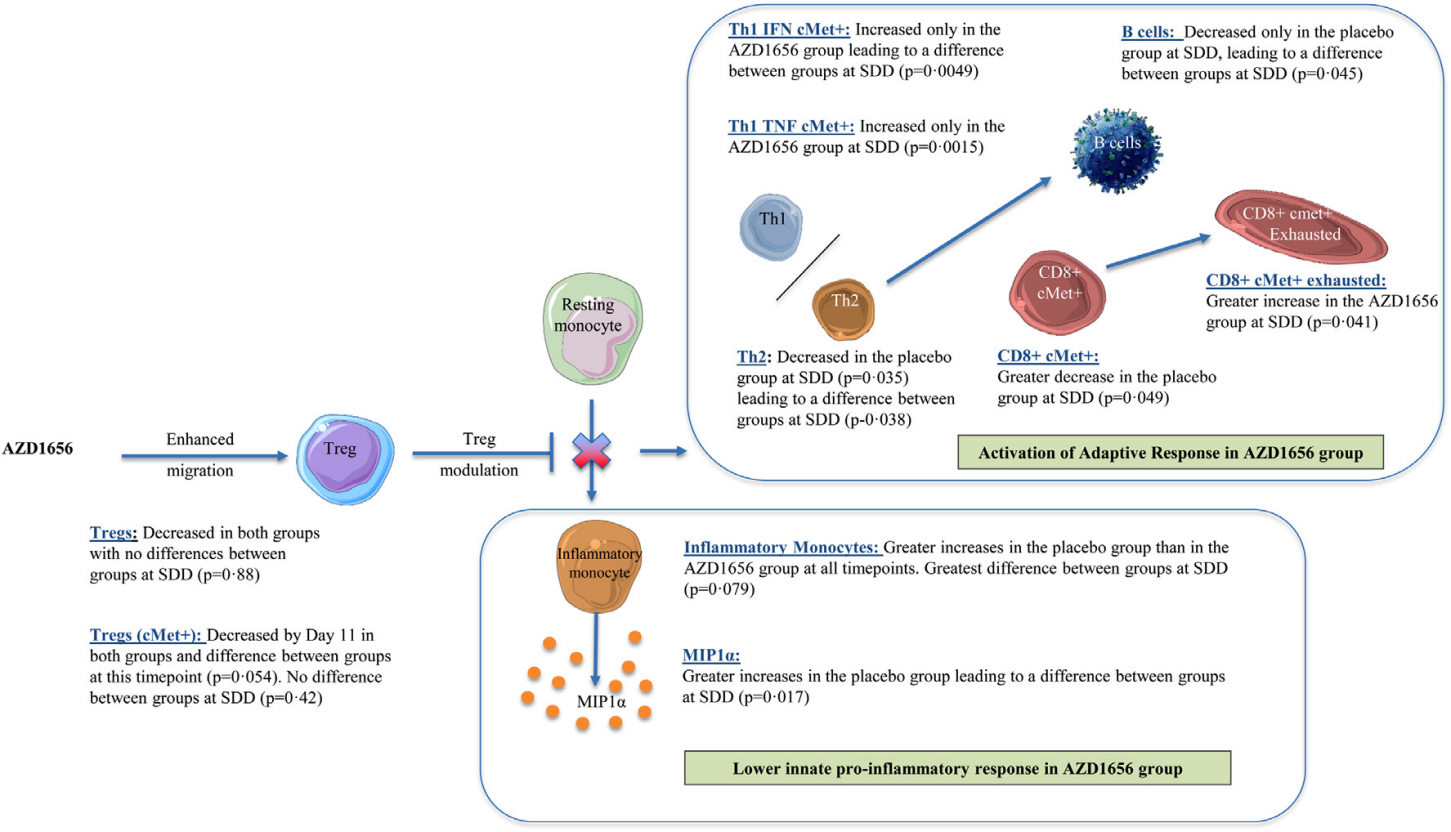
Immune effects of AZD1656 observed in the ARCADIA trial.

Immunophenotyping was consistent with AZD1656 playing a role in actively changing the immune response by moderating innate immunity, promoting the activation of an adaptive Th1 response and by maintenance of appropriate Th2 and B cell responses (Figure 6). A moderation of innate immunity was also observed in the immunochemistry data where a difference between groups in the pro-inflammatory cytokine MIP1α following treatment was seen. A reduction in activated Treg (cMet+) levels was seen at Day 11 only. Given that, at any time the Treg and Treg cMet+ count in the blood is the product of a dynamic equilibrium between cell production and cell use, the finding of a change in Treg (cMet+) at Day 11 might indicate a specific change in that equilibrium warranting further investigation.

A key strength of the trial was the simplicity of the study design, which allowed all new COVID-19 treatments to be given as they emerged during the pandemic. This encouraged a high level of engagement with investigators and patients and allowed the generation of data from normal clinical practice. Care was taken from the outset to design a trial which involved few interventions beyond those required for usual care, to minimize inconvenience to patients and to minimize additional burden to the investigators. As a result, we achieved our enrolment target with good patient retention and procedural study compliance throughout. A further strength of the trial was the use of electronic data capture which helped protocol compliance and team communication.

A key challenge was to design a clinical trial in a new disease that had not yet been characterised. This affected the choice and timing of the primary endpoint measure (clinical improvement as measured using the WHO OSCI rating system at Day 14) and the statistical power calculation, all of which were decided when there were no approved therapies for COVID-19. (It is worth noting that this issue applies to all trials designed in such circumstances and should encourage re-evaluation of the importance of post hoc analyses in clinical trials of emerging diseases). The prognosis for diabetic patients hospitalised with COVID-19 improved significantly through 2020 resulting in the majority of study patients being discharged from hospital prior to Day 14 in both groups. Whereas at the earlier Day 7 timepoint (identified a priori as a secondary endpoint measure for efficacy), the proportion of discharged patients was higher in the AZD1656 group than the Placebo group (by a ratio of 2:1) and there had been no deaths in the AZD1656 group compared to six in the placebo group. It is of pharmacological interest and potential clinical significance that the apparent therapeutic effect was achieved early in dosing, given that the mean duration of treatment was just over 7 days for all patients.

There have been significant advances in the development of therapeutic agents for COVID-19, but limitations continue to exist with currently approved therapies. Antiviral agents must be administered early in the infection, they can encourage viral mutation and use tends to be limited by toxicity. Monoclonal antibodies face logistical challenges and can be rendered ineffective by the emergence of new variants. We propose that AZD1656 would be an advance over other available therapeutics for COVID-19 if the results generated here are replicated in a larger confirmatory clinical trial. AZD1656 is a therapeutic intended specifically to treat the cellular disease itself caused by the virus, independent of any viral mutations which may occur.

Whilst the risk of false positives in a phase II trial exists, the ARCADIA trial data suggest that specific metabolic activation of Tregs brings about changes in the immune system which are associated with early clinical improvement in patients suffering inflammatory disease caused by a virus. Furthermore, the evidence presented here suggests that specific activation of Tregs might suppress the inappropriate inflammation which is the prime cause of tissue damage in autoimmune disease. If this is confirmed, further research into the possibility that cell activation may persist beyond the effect of the interaction of the small molecule and its intracellular molecular target would be warranted.

The ARCADIA trial has produced a new therapeutic concept: specific cell activation by a small molecule whereby the activated cell itself becomes the therapeutic agent within the body (“in vivo activated-cell therapy”). Although there have been some exceptions using embryonic cells,^21^ therapeutic cell therapy has, on the whole, failed.^22^^,^^23^ This is probably because until now in most cell therapy approaches cells have been removed from the body, processed and then reinjected; or cells have been cultured or engineered out of the body and then injected; rather than by therapeutic activation of the cell within its natural environment, as might be achieved with AZD1656. Furthermore, small molecule activators of Tregs could be used as pharmacological probes to reveal the role of Treg behaviour in conditions where cellular inflammation causes disease. These may be as diverse as the myocardial effects of ischaemia, premature labour, asthma, type 1 diabetes or sepsis.^24^, ^25^, ^26^, ^27^, ^28^ Although the trial was designed to address COVID-19, these findings might have wide implications for the development of new therapeutics across a spectrum of immune and other diseases and underscore the need for continued basic and clinical research in this area.

## Contributors

JC and JM conceived the study with input on trial design from DLL, KM, FMB and ZH. Management of the trial was overseen by JC and PB. Immunophenotyping was designed by FMB and conducted by CD and FMB. JM and DLL oversaw the interpretation of data with additional input and oversight from all other authors. JC, ZH and JM drafted the initial manuscript with input from FMB, KM, CD, PB and PC. JC and PB have verified the data. All authors had full access to all data and approved the final manuscript.

## Data sharing statement

The datasets analysed during the current study are not publicly available. Six months following article publication, researchers can request access to the datasets (including de-identified participant data and including data dictionaries), by providing a methodologically sound proposal. Proposals should be directed to the corresponding author. In addition, data requestors will need to sign a data access agreement to gain access. Once the proposal is approved, a link will be provided for access to the data at a third-party website.

## Declaration of interests

Dr. McCafferty reports grants and personal fees from AstraZeneca (who donated Drug Product for this trial) for activities outside of the submitted work. The authors declare no other competing interests.

## Appendix Supplementary materials

Image, application 1
