# Supplementary material for: A randomised, double-blind, placebo-controlled, multicentre clinical trial of AZD1656 in diabetic patients hospitalised with COVID-19: The ARCADIA Trial - implications for therapeutic immune modulation
Source: eClinicalMedicine. 2022 Aug 18;51:101604. doi: 10.1016/j.eclinm.2022.101604 (PMC9386394; doi:10.1016/j.eclinm.2022.101604)
Supplement: Supplementary file 1 [file mmc1.docx]

***Supplement 1:* Immunophenotyping Method used in the ARCADIA Trial**

The ARCADIA trial included an exploratory assessment of the effects of AZD1656 on immunophenotyping characteristics during COVID-19 infection in patients with diabetes and who had been admitted hospital with COVID-19. Queen Mary University of London (QMUL) conducted the immunophenotyping analysis for this trial, on behalf of the study sponsor St George Street Capital.

A blood sample for immunophenotyping analysis was collected at Visit 2 (Day 1), Visit 9 (Day 8), Visit 12 (Day 11) and Visit 22 (Day 21) and SDD. An immunophenotyping panel was conducted by flow cytometry to perform a group comparison (AZD1656 versus placebo) of levels of relevant immune cells (including T, DC, and B cell populations). See Table 1 for antibody panel details.

**Method**

Whole blood samples were collected from trial patients at the timepoints defined in the study protocol.  Samples were received at QMUL packed as per the Study Laboratory Manual. PBMCs were isolated from the whole blood using Ficoll Paque Plus (GE Healthcare, Cat# 17-1440-03) according to the manufacturers protocol. Total PBMCs were labelled for immunophenotyping using the antibody panels as shown in the table below. PBMCs were incubated in extracellular antibody cocktail mix for 18 minutes at 4^o^C, washed twice in MACs buffer, and centrifuged at 1900rpm for 3 minutes. If intracellular labelling was also required, cells were fixed in FOXP3 fix/perm buffer 4X (Biolegend, Cat# 421401) for 20 minutes at 4^o^C, washed once in FOXP3 perm buffer (Biolegend, #421402) and then incubated in the intracellular antibody mix for 18 minutes at 4^o^C. Finally cells were then washed once in perm buffer and once in MACs. All PBMCs were then fixed in BD stabilising fixative (BD Biosciences, #339860) and data acquired on the Beckman cytoFLEX LX flow cytometer.

| **Antibody** | **Source** | **Cat/lot. Number** |
| --- | --- | --- |
| AQUA live/dead | Invitrogen | Cat# L34966; 2309228 |
| Human Anti-CCR7 APC | Biolegend | Cat# 353214; B286358 |
| Human Anti-CD1c APC-CY7 | Biolegend | Cat# 331520; B285998 |
| Human Anti-CD123 PerCP | Biolegend | Cat# 306016; B25104 |
| Human Anti-CD127 PerCP | Biolegend | Cat# 351322; B293474 |
| Human Anti-CD14 APC | Biolegend | Cat# 325608; B288863 |
| Human Anti-CD141 PE-CY7 | Biolegend | Cat# 344110; B266383 |
| Human Anti-CD15 BV607 | Biolegend | Cat# 323032; B307528 |
| Human Anti-CD16 AF700 | Biolegend | Cat# 302026; B266048 |
| Human Anti-CD163 BV785 | Biolegend | Cat# 333632; B299825 |
| Human Anti-CD169 FITC | Invitrogen | Cat# 53-1699-42; 2150749 |
| Human Anti-CD19 BV605 | Biolegend | Cat# 302244; B281802 |
| Human Anti-CD27 SB645 | Invitrogen | Cat# 64-0279-42; 2162732 |
| Human Anti-CD3 APC-CY7 | Biolegend | Cat# 300318; B312625 |
| Human Anti-CD3 BV605 | Biolegend | Cat# 317322; B310622 |
| Human Anti-CD335 BV421 | Biolegend | Cat# 331914; B273928 |
| Human Anti-CD335 BV605 | Biolegend | Cat# 331926; B277544 |
| Human Anti-CD4 PE-CY5 | Biolegend | Cat# 317412; B272472 |
| Human Anti-CD4 PE-CY7 | Biolegend | Cat# 317414; B278098 |
| Human Anti-CD45 PE-CY5 | Biolegend | Cat# 304010; B284913 |
| Human Anti-CD45RO BV421 | Biolegend | Cat# 304224; B284688 |
| Human Anti-CD5 SB645 | Invitrogen | Cat# 64-0059-42; 2284086 |
| Human Anti-CD69 BV605 | Biolegend | Cat# 310938; B308115 |
| Human Anti-CD8 AF700 | Biolegend | Cat# 344724; B282429 |
| Human Anti-CD88 PECF594 | Biolegend | Cat# 344318; B268923 |
| Human Anti-CLEC9A PE | Biolegend | Cat# 353804; B273270 |
| Human Anti-c-Met FITC | Invitrogen | Cat# 11-8858-42; 2220927 |
| Human Anti-FOXP3 PE | Invitrogen | Cat# 12-4777-42; 2092018 |
| Human Anti-GARP PE-CY7 | Biolegend | Cat# 352508; B273169 |
| Human Anti-HLA-DR BV421 | Biolegend | Cat# 307636; B284694 |
| Human Anti-IFNγ APC | Biolegend | Cat# 502512; B279296 |
| Human Anti-IL-4 PECF594 | Biolegend | Cat# 500832; B281657 |
| Human Anti-IL-17 APC-CY7 | Biolegend | Cat# 512320; B279097 |
| Human Anti-KLRG1 BV785 | Biolegend | Cat# 138429; B307971 |
| Human Anti-PD-1 PECF594 | Biolegend | Cat# 329940; B285543 |
| Human Anti-TNFα BV785 | Biolegend | Cat# 502948; B275044 |
| ***Supplementary*** ***Table 1*: Antibodies used for Immunophenotyping** | | |

***Supplement 2*: Key Immunophenotyping Results from the ARCADIA Trial**

| Timepoint  Statistic | Placebo  (N = 73) | | AZD1656  (N = 80) | | AZD1656 vs. Placebo | |
| --- | --- | --- | --- | --- | --- | --- |
| Patients with data at Visit 9 (Day 8 +/- 1 day) | 28 | | 30 | | 58 | |
| Patient with data at Visit 12 (Day 9 +/- 1 day) | 15 | | 18 | | 33 | |
| Patients with data at SDD | 39 | | 49 | | 88 | |
| **Inflammatory Monocytes [%]** | |  | |  | |  |
| V9, mean (SD) | | 19·150 (15·003) | | 15·011 (13·897) | |  |
| Mean (SD) change from Baseline to V9 | | 8·163 (12·487) | | 1·872 (13·461) | |  |
| p-value | | 0·017 | | 0·97 | | 0·21 |
| V12, mean (SD) | | 24·142 (14·419) | | 16·310 (13·132) | |  |
| Mean (SD) change from Baseline to V12 | | 13·462 (15·239) | | 3·188 (15·830) | |  |
| p-value | | 0·0033 | | 0·64 | | 0·10 |
| SDD, mean (SD) | | 25·235 (16·220) | | 19·223 (13·356) | |  |
| Mean (SD) change from Baseline to SDD | | 14·054 (16·692) | | 4·153 (15·139) | |  |
| p-value | | < 0·0001 | | 0·058 | | 0·079 |
| **Dendritic Cells (DC) Percent [%]** | |  | |  | |  |
| V9, mean (SD) | | 7·023 (5·187) | | 7·505 (4·159) | |  |
| Mean (SD) change from Baseline to V9 | | -1·829 (6·735) | | -1·913 (5·945) | |  |
| p-value | | 0·15 | | 0·98 | | 0·25 |
| V12, mean (SD) | | 8·232 (9·613) | | 6·272 (2·604) | |  |
| Mean (SD) change from Baseline to V12 | | -0·329 (10·46) | | -4·112 (5·174) | |  |
| p-value | | 0·32 | | 0·36 | | 0·81 |
| SDD, mean (SD) | | 6·623 (3·621) | | 6·540 (6·382) | |  |
| Mean (SD) change from Baseline to SDD | | -2·677 (4·789) | | -2·525 (5·559) | |  |
| p-value | | 0·18 | | 0·0062 | | 0·20 |
| **Plasmacytoid Dendritic Cells (pDC) Percent [%]** | |  | |  | |  |
| V9, mean (SD) | | 1·2964 (1·550) | | 1·1232 (1·581) | |  |
| Mean (SD) change from Baseline to V9 | | -3·7227  (13·219) | | -2·7547 (6·247) | |  |
| p-value | | 0·0016 | | <0·0001 | | 0·42 |
| V12, mean (SD) | | 0·786 (0·779) | | 0·981 (1·56) | |  |
| Mean (SD) change from Baseline to V12 | | -7·961 (19·488) | | -5·330 (11·729) | |  |
| p-value | | 0·0012 | | 0·0002 | | 0·51 |
| SDD, mean (SD) | | 1·1528 (1·568) | | 1·7643 (6·118) | |  |
| Mean (SD) change from Baseline to SDD | | -3·2146 (12·031) | | -2·6312 (5·882) | |  |
| p-value | | < 0·0001 | | < 0·0001 | | 0·55 |
| **CD4+ T cells cMet+ [%]** |  | |  | |  | |
| V9, mean (SD) | 10·301 (12·135) | | 12·609 (16·283) | |  | |
| Mean (SD) change from Baseline to V9 | -4·633  (16·123) | | -6·236 (15·598) | |  | |
| p-value | 0·055 | | 0·054 | | 0·52 | |
| V12, mean (SD) | 11·629 (15·797) | | 8·449 (10·137) | |  | |
| Mean (SD) change from Baseline to V12 | -4·032 (19·483) | | -11·013 (16·200) | |  | |
| p-value | 0·15 | | 0·023 | | 0·60 | |
| SDD, mean (SD) | 9·390 (9·39) | | 9·815 (10·922) | |  | |
| Mean (SD) change from Baseline to SDD | -4·475  (12·495) | | -5·296 (15·764) | |  | |
| p-value | 0·017 | | 0·021 | | 0·91 | |
| **CD8+T cells cMet+ [%]** |  | |  | |  | |
| V9 Mean (SD) | 24·970 (16·878) | | 23·071 (18·74) | |  | |
| Mean (SD) change from Baseline to V9 | -1·411 (17·659) | | -6·479 (15·538) | |  | |
| p-value | 0·47 | | 0·41 | | 0·53 | |
| V12, mean (SD) | 21·289 (16·319) | | 16·187 (14·528) | |  | |
| Mean (SD) change from Baseline to V12 | -3·787 (18·881) | | -15·177 (15·066) | |  | |
| p-value | 0·20 | | 0·03 | | 0·30 | |
| SDD, mean (SD) | 21·627 (18·037) | | 22·022 (18·945) | |  | |
| Mean (SD) change from Baseline to SDD | -5·552 (17·943) | | -4·529 (22·256) | |  | |
| p-value | 0·049 | | 0·19 | | 0·94 | |
| **CD8+T cells Exhausted [%]** |  | |  | |  | |
| V9, mean (SD) | 9·058 (8·646) | | 14·112 (14·029) | |  | |
| Mean (SD) change from Baseline to V9 | 0·231 (14·104) | | 7·255 (15·635) | |  | |
| p-value | 0·21 | | 0·0019 | | 0·30 | |
| V12, mean (SD) | 5·770 (5·024) | | 10·898 (11·260) | |  | |
| Mean (SD) change from Baseline to V12 | -1·200 (14·668) | | 6·638 (13·197) | |  | |
| p-value | 0·95 | | 0·06 | | 0·33 | |
| SDD, mean (SD) | 11·333 (11·937) | | 10·183 (9·621) | |  | |
| Mean change from Baseline to SDD | 4·344 (10·492) | | 4·601 (9·402) | |  | |
| p-value | 0·028 | | 0·0036 | | 0·75 | |
| **CD8+T cells Exhausted cMet+ [%]** |  | |  | |  | |
| V9, mean (SD) | 31·681  (33·128) | | 28·791 (32·55) | |  | |
| Mean (SD) change from Baseline to V9 | 10·256 (39·394) | | 7·874 (36·243) | |  | |
| p-value | 0·74 | | 0·23 | | 0·69 | |
| V12, mean (SD) | 21·912 (21·912) | | 21·234 (28·243) | |  | |
| Mean (SD) change from Baseline to V12 | 8·712 (37·839) | | -2·560 (38·122) | |  | |
| p-value | 0·36 | | 0·87 | | 0·98 | |
| SDD, mean (SD) | 32·120 (32·686) | | 32·351 (32·033) | |  | |
| Mean (SD) change from Baseline to SDD | 3·105 (47·058) | | 6·994 (37·163) | |  | |
| p-value | 0·77 | | 0·041 | | 0·78 | |
| **Tregs [%]** |  | |  | |  | |
| V9, Mean (SD) | 16·071 (12·097) | | 25·382 (27·781) | |  | |
| Mean (SD) change from Baseline to V9 | -0·224 (10·768) | | -1·534 (16·030) | |  | |
| P value | 0·43 | | 0·82 | | 0·54 | |
| V12, mean (SD) | 15·756 (12·678) | | 18·001 (16·115) | |  | |
| Mean (SD) change from Baseline to V12 | 0·497 (8·269) | | -5·950 (18·488) | |  | |
| p-value | 0·50 | | 0·43 | | 0·86 | |
| SDD, mean (SD) | 17·810 (15·358) | | 17·510 (15·452) | |  | |
| Mean (SD) change from Baseline to SDD | -4·056 (14·661) | | -5·260 (15·143) | |  | |
| p-value | 0·53 | | 0·16 | | 0·88 | |
| **Tregs cMet+ [%]** |  | |  | |  | |
| V9, Mean (SD) | 18·449 (24·5164) | | 22·031 (29·2993) | |  | |
| Mean (SD) change from Baseline to V9 | -6·936 (27·998) | | 1·978 (29·119) | |  | |
| P value | 0·066 | | 0·38 | | 0·81 | |
| V12, mean (SD) | 32·589 (36·144) | | 8·901 (8·909) | |  | |
| Mean (SD) change from Baseline to V12 | -5·501 (25·033) | | -15·813 (28·327) | |  | |
| p-value | 0·88 | | 0·04 | | 0·05 | |
| SDD, mean (SD) | 25·653 (26·525) | | 22·192 (25·021) | |  | |
| Mean (SD) change from Baseline to SDD | -0·998 (31·457) | | -2·128 (28·242) | |  | |
| p-value | 0·59 | | 0·56 | | 0·41 | |
| **Th1-IFN [%]** |  | |  | |  | |
| V9, mean (SD) | 2·3468 (4·740) | | 3·4151 (9·443) | |  | |
| Mean (SD) change from Baseline to V9 | 0·4950 (4·249) | | 1·9784 (7·8274) | |  | |
| p-value | 0·046 | | 0·40 | | 0·89 | |
| V12, mean (SD) | 1·651 (1·883) | | 4·340 (10·645) | |  | |
| Mean (SD) change from Baseline to V12 | 0·7687 (1·847) | | 2·3180 (8·453) | |  | |
| p-value | 0·13 | | 0·09 | | 0·54 | |
| SDD, mean (SD) | 2·4097 (2·8924) | | 3·1873 (5·240) | |  | |
| Mean (SD) change from Baseline to SDD | 0·9439 (2·996) | | 1·5100 (4·435) | |  | |
| p-value | 0·0003 | | 0·016 | | 0·93 | |
| **Th1-IFN cMet+ [%]** |  | |  | |  | |
| V9, mean (SD) | 3·103 (10·256) | | 4·167 (18·465) | |  | |
| Mean (SD) change from Baseline to V9 | -6·026 (27·705) | | -0·286 (27·72) | |  | |
| p-value | 0·49 | | 0·46 | | 0·90 | |
| V12, mean (SD) | 1·407 (4·38) | | 3·310 (8·84) | |  | |
| Mean (SD) change from Baseline to V12 | -1·593 (6·64) | | -2·246 (25·26) | |  | |
| p-value | 0·94 | | 0·08 | | 0·36 | |
| SDD, mean (SD) | 0·276 (0·787) | | 5·457 (16·02) | |  | |
| Mean (SD) change from Baseline to SDD | -8·178 (27·049) | | 1·557 (24·255) | |  | |
| p-value | 0·97 | | 0·0006 | | 0·049 | |
| **Th1-TNF [%]** |  | |  | |  | |
| V9, mean (SD) | 21·861 (15·886) | | 23·448 (16·128) | |  | |
| Mean (SD) change from Baseline to V9 | -1·273 (12·965) | | 6·203 (11·97) | |  | |
| p-value | 0·34 | | 0·041 | | 0·77 | |
| V12, mean (SD) | 25·445 (16·782) | | 20·461 (18·839) | |  | |
| Mean (SD) change from Baseline to V12 | 0·286 (16·616) | | 1·154 (15·864) | |  | |
| p-value | 0·16 | | 0·63 | | 0·30 | |
| SDD, mean (SD) | 21·103 (13·994) | | 24·646 (16·481) | |  | |
| Mean (SD) change from Baseline to SDD | 1·552 (14·646) | | 3·806 (12·811) | |  | |
| p-value | 0·42 | | 0·0092 | | 0·24 | |
| **Th1-TNF cMet+ [%]** |  | |  | |  | |
| V9, Mean (SD) | 0·7239 (1·24255) | | 1·5443 (2·83642) | |  | |
| Mean (SD) change from Baseline to V9 | -3·1607 (10·379) | | 0·6472 (3·348) | |  | |
| p value | 0·83 | | 0·10 | | 0·61 | |
| V12, mean (SD) | 1·5873 (3·212) | | 2·7346 (3·902) | |  | |
| Mean (SD) change from Baseline to V12 | 0·4720 (3·732) | | 1·7252 (4·183) | |  | |
| p-value | 0·49 | | 0·0032 | | 0·29 | |
| SDD, mean (SD) | 1·1608 (2·011) | | 2·2394 (4·222) | |  | |
| Mean (SD) change from Baseline to SDD | -1·1326 (6·805) | | 1·2265 (4·285) | |  | |
| p-value | 0·16 | | 0·0015 | | 0·56 | |
| **Th2 [%]** |  | |  | |  | |
| V9, mean (SD) | 20·617 (17·015) | | 18·344 (10·577) | |  | |
| Mean (SD) change from Baseline to V9 | -5·836 (15·454) | | -5·513 (10·908) | |  | |
| p-value | 0·47 | | 0·85 | | 0·90 | |
| V12, mean (SD) | 24·415 (17·981) | | 26·158 (17·674) | |  | |
| Mean (SD) change from Baseline to V12 | -3·608 (16·853) | | -2·861 (16·203) | |  | |
| p-value | 0·79 | | 0·15 | | 0·63 | |
| SDD, mean (SD) | 15·994 (12·926) | | 21·224 (13·763) | |  | |
| Mean (SD) change from Baseline to SDD | -4·801 (17·314) | | -0·654 (14·713) | |  | |
| p-value | 0·035 | | 0·32 | | 0·038 | |
| **B Cells [%]** |  | |  | |  | |
| V9, mean (SD) | 23·715 (13·915) | | 23·715 (12·864) | |  | |
| Mean (SD) change from Baseline to V9 | 1·126 (10·625) | | 0·030 (15·285) | |  | |
| p-value | 0·87 | | 0·70 | | 0·95 | |
| V12, mean (SD) | 21·048 (12·604) | | 22·747 (12·561) | |  | |
| Mean (SD) change from Baseline to V12 | -3·249  (8·855) | | -0·604 (9·638) | |  | |
| p-value | 0·55 | | 0·96 | | 0·53 | |
| SDD, mean (SD) | 19·571 (12·103) | | 25·001 (13·408) | |  | |
| Mean (SD) change from Baseline to SDD | -4·910 (14·009) | | 0·944 (12·789) | |  | |
| p-value | 0·22 | | 0·31 | | 0·045 | |
| Comparisons to baseline values are shown for each treatment group and between groups at timepoints: V9 (Day 8 +/- 1 day), V12 (Day 11 +/- 1 day) and SDD (Study Drug Discontinuation). SDD could occur on any timepoint between D2 and D21.  N = total number of patients; IFN = interferon; TNF = tumour necrosis factor | | | | | | |
| ***Supplementary Table 2*: Key immunophenotyping results** | | | | | | |

***Supplement 3*: Key Immunochemistry Results from the ARCADIA Trial**

| Timepoint  Statistic | Placebo  (N = 73) | AZD1656  (N = 80) | AZD1656 vs. Placebo |
| --- | --- | --- | --- |
| Patients with data at Visit 9 (Day 8 +/- 1 day) | 30 | 30 | 60 |
| Patients with data at Visit 12 (Day 11 +/- 1 day) | 17 | 18 | 35 |
| Patients with data at SDD | 38 | 51 | 89 |
| **MIP1α [pg/mL]** |  |  |  |
| V9, mean (SD) | 34·24 (14·650) | 36·01 (24·189) |  |
| Mean (SD) change from Baseline to V9 | 4·57 (18·065) | 4·57 (23·272) |  |
| p-value | 0·11 | 0·01 | 0·64 |
| V12, mean (SD) | 31·35 (14·704) | 35·31 (18·876) |  |
| Mean (SD) change from Baseline to V12 | 2·54 (15·426) | 4·78 (20·851) |  |
| p-value | 0·39 | 0·02 | 0·91 |
| SDD Mean (SD) | 35·72 (19·667) | 28·02 (13·664) |  |
| Mean (SD) change from Baseline to SDD | 6·49 (17·9) | 1·33 (15·039) |  |
| p-value | 0·052 | 0·15 | 0·017 |
| **GCSF [pg/mL]** |  |  |  |
| V9, mean (SD) | 10·621 (6·238) | 199·333 (1037·036) |  |
| Mean (SD) change from Baseline to V9 | -9·692 (16·088) | 181·031 (1037·454) |  |
| p-value | 0·0036 | 0·0001 | 0·98 |
| V12, mean (SD) | 10·839 (78·26) | 9·253 (4·339) |  |
| Mean (SD) change from Baseline to V12 | -13·215 (17·45) | -9·782 (11·426) |  |
| p-value | 0·022 | < 0·0001 | 0·77 |
| SDD, mean (SD) | 12·158 (6·537) | 11·938 (12·453) |  |
| Mean (SD) change from Baseline to SDD | -8·994 (16·425) | -7·675 (16·900) |  |
| p-value | 0·028 | < 0·0001 | 0·17 |
| **Interleukin 10 [pg/mL]** |  |  |  |
| V9, mean (SD) | 1·774 (2·454) | 1·948 (2·396) |  |
| Mean (SD) change from Baseline to V9 | -1·785 (4·077) | -1·601 (4·397) |  |
| p-value | 0·0025 | 0·018 | 0·38 |
| V12, mean (SD) | 1·090 (0·598) | 1·042 (0·721) |  |
| Mean (SD) change from Baseline to V12 | -2·018 (2·941) | -2·738 (4·668) |  |
| p-value | 0·0023 | 0·0009 | 0·59 |
| SDD, mean (SD) | 1·826 (3·501) | 1·384 (2·051) |  |
| Mean (SD) change from Baseline to SDD | -1·586 (4·672) | -1·746 (4·659) |  |
| p-value | 0·0006 | < 0·0001 | 0·22 |
| Comparisons to baseline values are shown for each treatment group and between groups at timepoints: V9 (Day 8 +/- 1 day), V12 (Day 11 +/- 1 day) and SDD (Study Drug Discontinuation). SDD could occur on any timepoint between D2 and D21.  N = total number of patients; MIP1α = macrophage inflammatory protein 1α; GCSF = granulocyte colony stimulating factor | | | |
| ***Supplementary Table 3*: Key immunochemistry results** | | | |

***Supplementary Table 4.*  Summary of Concomitant Medications (either reported in ≥ 30% of patients, or of special interest in COVID-19).**

| ATC Level 4  Generic name | Placebo  (N = 73) | AZD1656  (N = 80) | All patients  (N = 153) |
| --- | --- | --- | --- |
| At least one medication | 73 (100%) | 80 (100%) | 153 (100%) |
|  |  |  |  |
| ACE inhibitors, plain | 26 (35.6%) | 26 (32.5%) | 52 (34.0%) |
| Anilides | 32 (43.8%) | 39 (48.8) | 71 (46.4%) |
| Paracetamol | 32 (43.8%) | 38 (47.5) | 70 (45.8%) |
| Beta blocking agents, selective | 27 (37.0%) | 21 (26.3%) | 48 (31.4%) |
| Biguanides | 45 (61.6%) | 46 (57.5%) | 91 (59.5%) |
| Metformin | 34 (46.6%) | 34 (42.5%) | 68 (44.4%) |
| Metformin hydrochloride | 12 (16.4%) | 13 (16.3%) | 25 (16.3%) |
| Glucocorticoids | 59 (80.8%) | 61 (76.3%) | 120 (78.4%) |
| Dexamethasone | 55 (75.3%) | 57 (71.3%) | 112 (73.2%) |
| Heparin group | 53 (72.6%) | 61 (76.3%) | 114 (74.5%) |
| HMG CoA reductase inhibitors | 39 (53.4%) | 38 (47.5%) | 77 (50.3%) |
| Insulins and analogues for injection, fast-acting | 27 (37.0%) | 30 (37.5%) | 57 (37.3%) |
| Proton pump inhibitors | 49 (67.1%) | 41 (51.3%) | 90 (58.8%) |
| Solutions affecting the electrolyte balance | 26 (35.6%) | 28 (35.0%) | 54 (35.3%) |
| Interleukin Inhibitors | 3 (4.1%) | 5 (6.3%) | 8 (5.2%) |
| Tocilizumab | 3 (4.1%) | 3 (3.8%) | 6 (3.9) |
| Anakinra | 0 | 1 (1.3%) | 1 (0.7) |
| Sarilumab | 0 | 1 (1.3%) | 1 (0.7) |
| ACE = angiotensin converting enzyme; ATC= Anatomical Therapeutic Chemical; FAS = full analysis set; N = number of patients in the analysis set. | | | |
| ***Supplementary Table 4.*  Summary of Concomitant Medications (either reported in ≥ 30% of patients, or of special interest in COVID-19).** | | | |

***Supplementary Figure 1.*  Time From Randomisation to Hospital Discharge by Vitamin D Level at Baseline - post hoc (Analysis Set: FAS)**
